# Calcium Phosphate Nanoclusters for the Repair of Tooth Enamel Erosion

**DOI:** 10.3390/nano12121997

**Published:** 2022-06-10

**Authors:** Chia-Hsien Wang, Chinmaya Mutalik, Sibidou Yougbaré, Nai-Chia Teng, Tsung-Rong Kuo

**Affiliations:** 1 Division of Prosthodontic Dentistry, Department of Dentistry, Taipei Medical University Hospital, Taipei 11031, Taiwan; 133013@h.tmu.edu.tw; 2 International Ph.D. Program in Biomedical Engineering, College of Biomedical Engineering, Taipei Medical University, Taipei 11031, Taiwan; d845108002@tmu.edu.tw; 3 Institut de Recherche en Sciences de la Santé/Direction Régionale du Centre Ouest (IRSS/DRCO), Nanoro BP 218, 11, Burkina Faso; ysibidou@gmail.com; 4 School of Dentistry, College of Oral Medicine, Taipei Medical University, Taipei 11031, Taiwan; 5 Dental Department, Taipei Medical University Hospital, Taipei 11031, Taiwan; 6 Graduate Institute of Nanomedicine and Medical Engineering, College of Biomedical Engineering, Taipei Medical University, Taipei 11031, Taiwan

**Keywords:** repair, calcium phosphates, tooth enamel, nanocluster, enamel rod, microhardness test

## Abstract

The artificial repair of tooth enamel is still an urgent requirement because it has a complicated and well-arranged structure. Herein, calcium phosphate nanoclusters (CaP NCs) were synthesized, via a facile approach, for application in the repair of tooth enamel erosion. Structural and optical characterizations validated the successful preparation of spherical CaP NCs, with an average size of 2.1 ± 0.11 nm. By evaporating the ethanol and triethylamine (TEA) solvents, pure CaP was produced, which was further used to repair the tooth enamel. Simulated caries lesions were achieved via phosphoric acid etching to cause damage to enamel rods. After repair, the damaged enamel rods were directly covered with CaP. According to microhardness testing, after repair with CaP NCs, the hardness value of the tooth enamel with acid etching increased to a similar level to that of normal tooth enamel. The results of the microhardness test indicated that CaP NCs revealed great potential for repairing tooth enamel erosion. Our work demonstrates a promising potential for treating the early stage of tooth erosion with CaP NCs. Based on these findings, we believe that stable CaP NCs can be employed as a precursor for the tunable, effective repair of tooth enamel in the near future.

## 1. Introduction

The enamel on the outer surface layer of the teeth is the hardest mineral substance in the human body, and it protects against tooth erosion from dietary acids and toxins released by plaque bacteria [1,2,3,4,5,6,7,8,9]. However, mature enamel is dead tissue and, therefore, cannot be naturally regenerated after erosion. Tooth decay is also known as the most common chronic disease in the world [10,11,12]. Although tooth enamel cannot be naturally repaired, several approaches have been utilized to artificially repair tooth enamel, including dental bonding, veneers, crowns, and remineralization [13,14,15,16,17,18,19]. For dental bonding, a dental resin is attached to the tooth surface to repair the enamel erosion and protect the intact tooth surface [20]. Porcelain veneers are employed to restore severe damage to the enamel on the front of a tooth [21]. In the worst cases of erosion of the enamel, damaged teeth are repaired with dental crowns to cover their entire surface [22]. With slight damage, the enamel can be restored via toothpaste treatment due to remineralization with calcium and essential minerals in the toothpaste [23]. The above approaches provide alternative treatments for the common, chronic disease of tooth decay.

Nanomaterials synthesized by metals, semiconductors, and oxides with distinctive chemical and physical characteristics have been extensively explored for applications in biomedicine, energy transformation, and electronics [24,25,26,27,28,29,30,31,32,33,34,35,36,37,38]. In various nanomaterials, nanoclusters (NCs) with sizes ranging from several atoms to a few hundred atoms have garnered great attention because of their superior structural and optical behaviors [39,40,41,42,43,44]. For example, cysteine-conjugated gold NCs were applied for bacterial detection and inhibition due to their unique size and fluorescence [39]. Furthermore, gold NCs with a surface coating of glutathione were used as intracellular, biocompatible light absorbers to overcome the sluggish kinetics of electron transfer in an artificial photosynthetic biohybrid system [45]. [Au_25_(PPh_3_)_10_(SC_2_H_4_Ph)_5×2_]^2+^ NCs with optimal cluster size and organic ligands revealed semiconducting behavior, electric field effects, and photoconductivity [46]. Ultra-small copper NCs, including [Cu_3_(μ_3_-H)(μ_2_-dppy)_4_](ClO_4_)_2_ and [Cu_4_(μ_4_-H)(μ_2_-dppy)_4_(μ_2_-Cl)_2_](ClO_4_), were found to have ultra-bright yellow and yellowish-green room-temperature phosphorescence emissions, and yellow and white light-emitting diodes were also fabricated and characterized [47]. These great achievements prove the promising potential for nanotechnological applications of NCs.

The repair of tooth enamel is still a difficult challenge because of its complicated and well-arranged structure. Calcium phosphate has been demonstrated as the biomineralization frontier for inducing the epitaxial regeneration of enamel [48]. In this work, NCs composed of calcium and phosphate (CaP NCs) were synthesized as a precursor to repairing tooth enamel. The CaP NCs were prepared via a simple approach. Transmission electron microscopy (TEM), energy-dispersive X-ray (EDX) spectroscopy, and Fourier-transform infrared (FTIR) spectroscopy were used to characterize the structural and optical properties of the CaP NCs. Furthermore, the ability of the CaP NCs to repair tooth enamel was investigated. Scanning electron microscopy (SEM) and a microhardness test were utilized to examine the tooth enamel before and after repair with CaP NCs.

## 2. Materials and Methods

### 2.1. Chemicals

Calcium chloride dihydrate (CaCl_2_·2H_2_O, ACS reagent, ≥99%), triethylamine (TEA, (C_2_H_5_)_3_N, ≥99.5%), ethanol (C_2_H_5_OH), and phosphoric acid (H_3_PO_4_, ACS reagent, ≥85 wt% in H_2_O) were purchased from Sigma-Aldrich (St. Louis, MO, USA) and used without further purification.

### 2.2. Preparation of CaP NCs via a Facile Approach

CaP NCs were prepared via a facile method according to the previous literature, with some modifications [48]. To synthesize CaP NCs, precursor solutions of A and B were first prepared. To prepare precursor solution A, 1.4 mmol of CaCl_2_·2H_2_O and 3.8 mL of TEA were added to 80 mL of ethanol under ultrasonication. To prepare precursor solution B, a 70 μL solution of H_3_PO_4_ was added to 20 mL of ethanol under vigorous stirring. Afterward, precursor solution B was added to precursor solution A under vortexing (Vortex-Genie 2, Scientific Industries, Bohemia, NY, USA). After being vortexed, a cloudy solution with CaP NCs was obtained. The solution containing CaP NCs was purified via centrifugation. After centrifugation, the CaP NC precipitate was redispersed in ethanol. The purification processes were repeated twice. The final concentration of CaP NCs in ethanol was 2.2 mg/mL.

### 2.3. Preparation of Tooth Samples

Human wisdom teeth samples were disinfected in an ethanol solution (70%, *v*/*v*) at room temperature for 48 h. After disinfection, a tooth was embedded in resin. The tooth embedded in resin was then dried in a chemical fume hood with air flow for 24 h. After drying for 24 h, the tooth embedded in resin was cut in half with a low-speed diamond saw. Afterward, the sample of the tooth embedded in resin was washed with deionized water. To create a tooth enamel window (3 cm^2^), the resin on the tooth surface was ground and polished with a grinder (PM2-200SA, PlusOver, Kaohsiung, Taiwan). The tooth with the enamel window was washed with deionized water and then dried in air for the following experiments.

### 2.4. Repair of the Tooth Enamel with CaP NCs

To simulate caries lesions, a tooth enamel window was treated with H_3_PO_4_ (37 wt% in H_2_O) various times. After acid etching, the tooth was washed with deionized water under ultrasonication to eliminate impurities, and then the tooth sample was dried in air. Before repair of the tooth enamel with CaP NCs, half of the enamel window was protected by nail varnish. The other half of the enamel window was repaired with CaP NCs. The solution of CaP NCs was dropped onto the portion of the enamel window, which was not protected with nail varnish. Afterward, the tooth sample was dried in air at 25 °C for 48 h. After 48 h, to allow the CaP NCs to crystallize on the enamel, the tooth sample was washed with deionized water for 20 min under ultrasonication and then dried at 25 °C in air for 24 h. The tooth sample was stored at 25 °C in air for further characterization.

### 2.5. Material Characterization

TEM and SEM with EDX were performed with HT-7700 (Hitachi, Tokyo, Japan) and SU3500 (Hitachi, Tokyo, Japan) instruments, respectively. The TEM sample of CaP NCs was sonicated in ethanol and then dropped onto a copper mesh, dried, and applied for imaging. FTIR spectroscopy (Thermo Scientific Nicolet iS10, Waltham, MA, USA) was applied to investigate the functional groups of CaP NCs. A microhardness tester (HMV-2, Shimadzu, Tokyo, Japan) was utilized to evaluate the hardness of the tooth enamel.

### 2.6. Microhardness Test

For microhardness testing, an indentation was made in the tooth enamel with a diamond indenter. The length of the indentation was measured, and then the following equation was applied to calculate the Vickers hardness value (HV), where F is the applied load (N), and d is the average diagonal length (mm) of the indentation:

HV = 0.891 × F/d^2^ and

d = (d_1_ + d_2_)/2

## 3. Results and Discussion

### 3.1. Characterizations of CaP NCs

**Figure 1:**
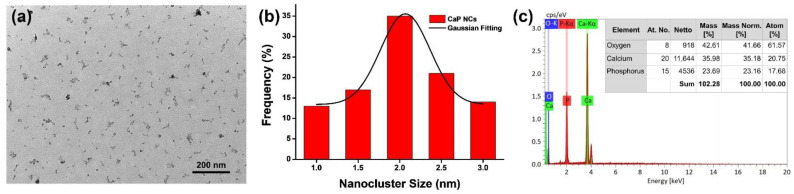
(**a**) TEM image of calcium phosphate nanoclusters (CaP NCs). (**b**) Histogram of the size distribution of CaP NCs and its Gaussian fitting curve. (**c**) EDX analysis of CaP NCs.

TEM was utilized to characterize the morphology of CaP NCs. As shown in Figure 1a, the CaP NCs revealed an approximately spherical shape. Furthermore, the CaP NCs showed a homogenous distribution on the copper grid without aggregation. To calculate the average size, the size distribution of CaP NCs was determined. Based on the statistical calculation of 100 NCs in the TEM image of Figure 1a, a histogram of the size distribution of CaP NCs and its simulated Gaussian fitting curve are shown in Figure 1b. The average size of CaP NCs was calculated to be 2.1 ± 0.2 nm using the Gaussian fitting curve. Moreover, EDX was applied to analyze the composition of CaP NCs. As shown in Figure 1c, CaP NCs were composed of calcium (35.18 wt%), phosphorus (23.16 wt%), and oxygen (41.66 wt%). The EDX analysis demonstrated that CaP NCs were composed of calcium and phosphate. Overall, the characterizations including TEM images and EDX analytical results demonstrated the successful synthesis of CaP NCs via a facile approach.

### 3.2. Stability Evaluation of CaP NCs in TEA

**Figure 2:**
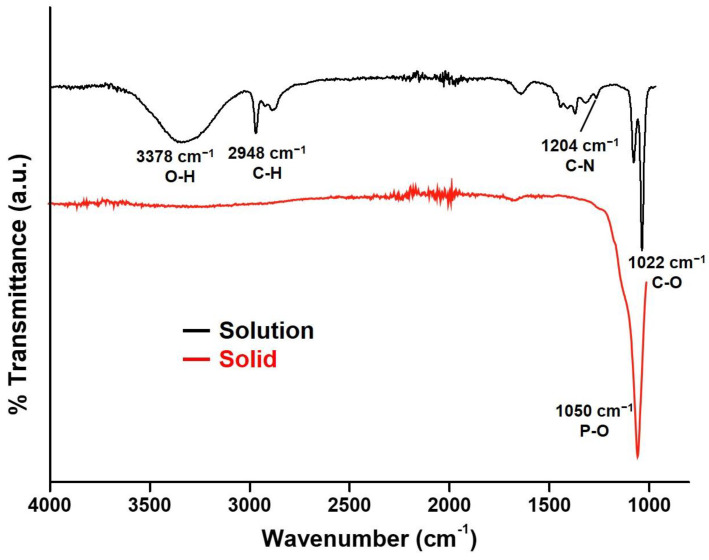
FTIR spectra of the calcium phosphate nanocluster (CaP NC) solution and solid.

Ultrasmall NCs can very easily form aggregates or increase in size. Herein, TEA was applied to prevent CaP NCs from aggregating because of the binding between TEA and the phosphate of CaP NCs [48]. FTIR was utilized to examine the binding between TEA and the phosphate of CaP NCs. As shown in Figure 2, the FTIR spectrum of a CaP NC solution (black curve) exhibited characteristic absorption peaks of ethanol at 1022 (C-O stretching), 2948 (C-H stretching), and 3378 cm^−1^ (O-H stretching), due to the ethanol solvent. Most importantly, the stretching (1204 cm^−1^ of C-N) of TEA indicated the existence of TEA in the CaP NC solution. The C-N stretching of TEA is typically located at 1200 cm^−1^ in an ethanol solution [48]. The C-N stretching shifted from 1200 to 1204, which can be attributed to an interaction between TEA and CaP NCs, resulting in the stabilization of CaP NCs by TEA, corresponding to the observation in TEM images. After evaporating the ethanol and TEA, the FTIR spectrum of CaP NC solids (red curve) revealed a characteristic absorption peak at 1050 cm^−1^ (P-O stretching) because of CaP. The complete removal of ethanol and TEA indicated that CaP NCs could form pure CaP for the further repair of tooth enamel.

### 3.3. Development of CaP NCs for Repairing Tooth Enamel

**Figure 3:**
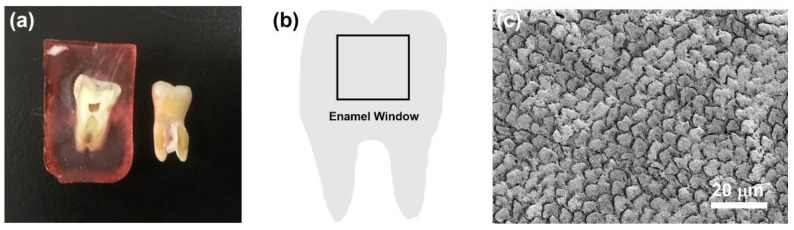
(**a**) Photo of tooth embedded in resin (**left**) and removed from the resin (**right**). (**b**) Illustration of a tooth enamel window. (**c**) SEM image of the tooth enamel before acid etching.

To demonstrate the repair of tooth enamel with CaP NCs, a tooth sample was prepared. As shown in Figure 3a, the tooth was embedded in resin (left), and then the resin was removed (right). To simulate a caries lesion, a tooth enamel window was created on the surface of the tooth sample, as shown in the illustration of Figure 3b. The tooth enamel window was further characterized with SEM before acid etching. As shown in the SEM image of Figure 3c, well-aligned and intact enamel rods were interwoven with inter-rods to reveal a unique fish-scale-shaped structure.

**Figure 4:**
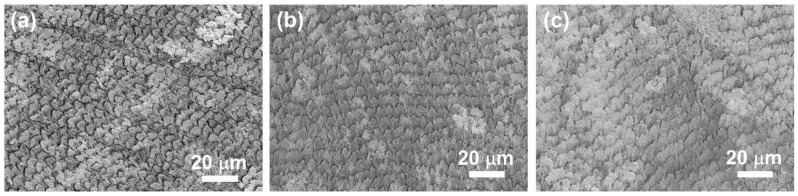
SEM images of tooth enamel after acid etching for (**a**) 30 s, (**b**) 10 min, and (**c**) 20 min.

To simulate a caries lesion, the tooth enamel window was etched with phosphoric acid for various time durations, including 0.5, 10, and 20 min. As shown in Figure 4a, with acid etching for 30 s, the tooth enamel showed no significant change compared to tooth enamel before acid etching. However, after acid etching for 10 min, the enamel rods revealed obvious damage on their tops, as shown in Figure 4b. Furthermore, after acid etching for 20 min, the enamel rods exhibited serious destruction of the entire enamel rods, as shown in Figure 4c. To further investigate the repair of tooth enamel with CaP NCs, a sample of tooth enamel with 10 min of acid etching was selected, due to the minor damage to its tooth enamel.

**Figure 5:**
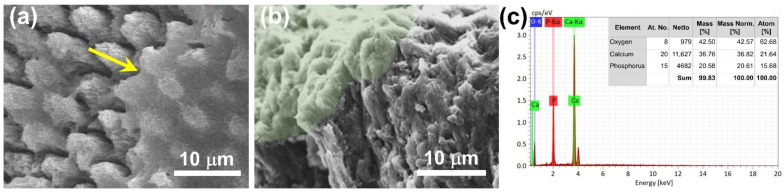
SEM image of tooth enamel after repair with calcium phosphate nanoclusters (CaP NCs). (**a**) The yellow arrow indicates the enamel rods covered with CaP. (**b**) Cross-sectional SEM image of the tooth enamel after repair with CaP NCs. (**c**) EDX analysis of the elemental composition of tooth enamel after repair with CaP NCs.

The tooth enamel sample with 10 min of acid etching was characterized with SEM after being further repaired with CaP NCs. In the SEM image of Figure 5a, the enamel rods were covered with CaP as indicated by the yellow arrow. As shown in a cross-sectional SEM image of Figure 5b, the region with a light-green color revealed that CaP grew directly on the top of the tooth enamel. Furthermore, as shown in Figure 5c, EDX analysis revealed that the elemental compositions of tooth enamel were composed of calcium (36.82 wt%), phosphorus (20.61 wt%), and oxygen (42.57 wt%) after repair with CaP NCs. The EDX analyses indicated that the elemental compositions of CaP NCs were similar in comparison with those of tooth enamel after repair with CaP NCs. Overall, the results indicated that the repair of tooth enamel with CaP NCs is a promising approach to treating the early stage of tooth erosion.

### 3.4. Microhardness Test of the Tooth Enamel before and after Repair with CaP NCs

**Figure 6:**
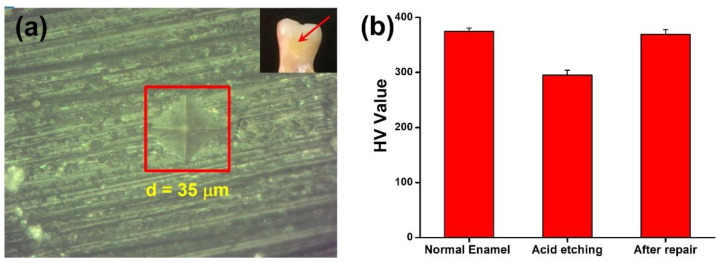
(**a**) Photo of indentation on the tooth enamel indicated by a red square. The length of indentation was 35 μm. The inset indicates the location of indentation. (**b**) Vickers hardness values of normal tooth enamel, tooth enamel after acid etching for 10 min, and tooth enamel after acid etching and repair with CaP NCs. All data are reported as the mean ± standard deviation, with *n* = 5 per category.

A microhardness test is commonly used to measure hardness in the microstructural phase or surface layer. In this work, a microhardness test was utilized to evaluate the hardness of the tooth enamel in different situations, including normal, after acid etching, and after repair with CaP NCs. As shown in Figure 6a of microhardness testing, the length of the indentation was measured, and then the value of HV was calculated. HVs of normal tooth enamel, tooth enamel after acid etching for 10 min, and tooth enamel after acid etching and repair with CaP NCs were, respectively, calculated to be 374.43, 294.85, and 368.97 HV, as shown in Figure 6b. Compared to the normal tooth enamel, the hardness of tooth enamel with acid etching decreased because of the destruction of the intact enamel. After repair with CaP NCs, the hardness value of the tooth enamel with acid etching increased to a similar level to that of normal tooth enamel. Results of the microhardness test indicated that CaP NCs revealed great potential for the repair of tooth enamel erosion.

## 4. Conclusions

CaP NCs were prepared via a facile approach, and their structural and optical characterizations demonstrated successful preparation. To avoid aggregation, TEA was applied to bind with the phosphate of the CaP NCs. Furthermore, CaP NCs were utilized as a precursor to repairing tooth enamel erosion. To simulate a caries lesion, the tooth enamel was etched with phosphoric acid for various time durations. The tops of the enamel rods revealed obvious damage, due to acid etching for 10 min, and the entire enamel rods exhibited serious destruction after acid etching for 20 min. The tooth enamel sample with 10 min of acid etching was further repaired with CaP NCs. After repair, the enamel rods were covered with CaP. In a cross-sectional SEM image, CaP was seen to have grown directly onto the top of the tooth enamel. The microhardness test indicated that CaP NCs can repair tooth enamel erosion. Overall, in this work, the repair of tooth enamel with CaP NCs revealed a promising potential for treating the early stage of tooth erosion.

## Data Availability

The data presented in this study are available on request from the corresponding author.
